# Minimally Invasive Coronary Artery Bypass With Composite Arterial Graft for High-Bifurcation Anomalous Aortic Origin of Right Coronary Artery

**DOI:** 10.7759/cureus.106666

**Published:** 2026-04-08

**Authors:** George Varghese Kurien, Lekshmipriya Govind J, Glen Loui Raphy, Manoj P Nair, Sunil Roy Thottuvelil Narayanan

**Affiliations:** 1 Cardiothoracic and Vascular Surgery, Aster Institute of Cardiac Sciences, Aster Medcity, Kochi, IND; 2 Anesthesiology and Critical Care, Aster Medcity, Kochi, IND; 3 Clinical Research, Aster Institute of Cardiac Sciences, Aster Medcity, Kochi, IND; 4 Interventional Cardiology, Aster Institute of Cardiac Sciences, Aster Medcity, Kochi, IND

**Keywords:** aaorca, composite arterial graft, midcab, right internal mammary artery, sudden cardiac death

## Abstract

Anomalous aortic origin of the right coronary artery (AAORCA) following an interarterial path can lead to myocardial ischemia and sudden cardiac death (SCD), necessitating surgical management in patients with symptoms. We present a case of a 35-year-old male experiencing exertional angina with reduced functional capacity. Computed tomography coronary angiography (CTCA) demonstrated the right coronary artery (RCA) arising from the left coronary cusp, characterized by a brief intramural segment, dangerous interarterial course, and elevated bifurcation creating dual RCA/posterior descending artery (PDA) branches. The intricate branching anatomy, combined with the abbreviated intramural portion, made unroofing an unfavorable option. We performed a right-sided minimally invasive direct coronary artery bypass (MIDCAB) employing a right internal mammary artery (RIMA)-radial artery composite graft to the PDA, accompanied by proximal native vessel ligation. Successful revascularization was achieved with graft patency confirmed on postoperative imaging, absence of myocardial infarction, and smooth recovery leading to discharge on postoperative day two. This experience suggests that composite arterial grafting through a minimally invasive technique provides effective symptom resolution in anatomically challenging AAORCA cases where conventional surgical approaches are not optimal.

## Introduction

Anomalous aortic origin of the right coronary artery (AAORCA) is a rare congenital abnormality in which the right coronary artery (RCA) arises from an atypical aortic location and follows an aberrant course, resulting in a wide spectrum of clinical manifestations ranging from incidental detection to myocardial ischemia, malignant arrhythmias, and sudden cardiac death (SCD) [1]. We demonstrate a successful case of right-sided minimally invasive direct coronary artery bypass (MIDCAB) - a technique that achieves coronary revascularization through a small thoracotomy without full sternotomy - for a high-bifurcation AAORCA.

## Case presentation

**Figure 1:**
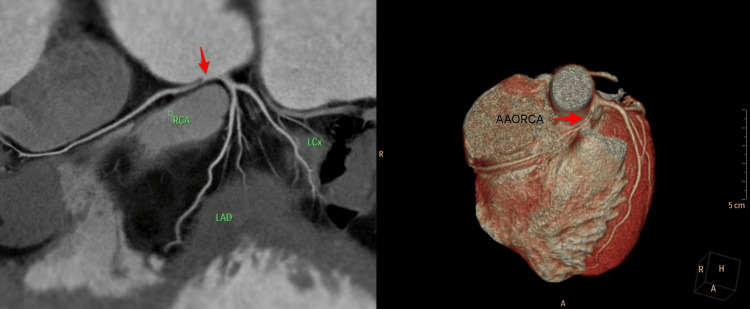
Preoperative computed tomography coronary angiography with reconstruction showing right coronary arising from left coronary cusp with short intramural course and long interarterial course. RCA: right coronary artery, LAD: left anterior descending, LCx: left circumflex, AAORCA: anomalous aortic origin of the right coronary artery

A 35-year-old male with no known comorbidities presented with exertional angina for one month. His functional capacity was poor, with metabolic equivalents <4, and a treadmill stress test (TMT) could not be completed due to dyspnoea and ongoing ST-T changes following which the TMT was terminated preemptively. Transthoracic echocardiography demonstrated good biventricular function. Stress electrocardiogram (ECG) was inconclusive due to poor exercise tolerance. Computed tomography coronary angiography (CTCA) revealed an anomalous origin of the RCA from the left coronary cusp, adjacent to the left main ostium, with a short intramural course and a malignant interarterial course between the aorta and pulmonary artery before reaching the right atrioventricular groove with a high bifurcation into a dual RCA/early posterior descending artery (PDA). The left anterior descending (LAD) and left circumflex (LCx) arteries were normal, with a calcium score of zero (Figure 1). 

Given the patient’s symptoms and high-risk anatomy, surgical intervention was planned. We opted for a minimally invasive approach in view of the peculiar anatomy of the already anomalous coronary artery with high early bifurcation and purely interarterial course and patient preference for minimal invasive surgery. The planned procedure was MIDCAB using a right internal mammary artery (RIMA)-radial artery composite graft to the PDA on beating-heart support with a pledgetted plication of proximal RCA.

**Figure 2:**
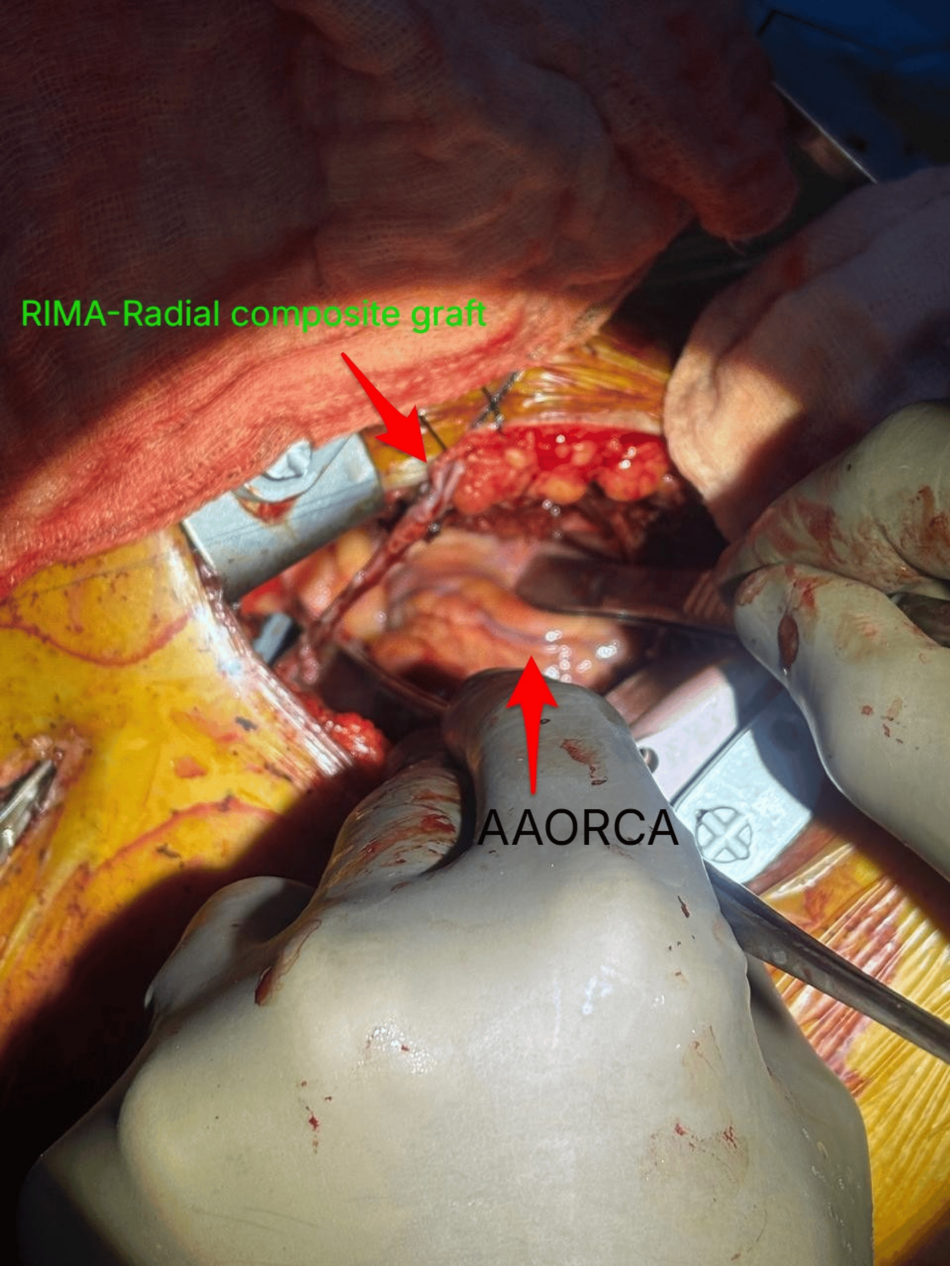
RIMA-radial composite graft prepared. High early posterior descending artery bifurcation shown in background where forceps is pointed. RIMA: right internal mammary artery, AAORCA: anomalous aortic origin of the right coronary artery

**Figure 3:**
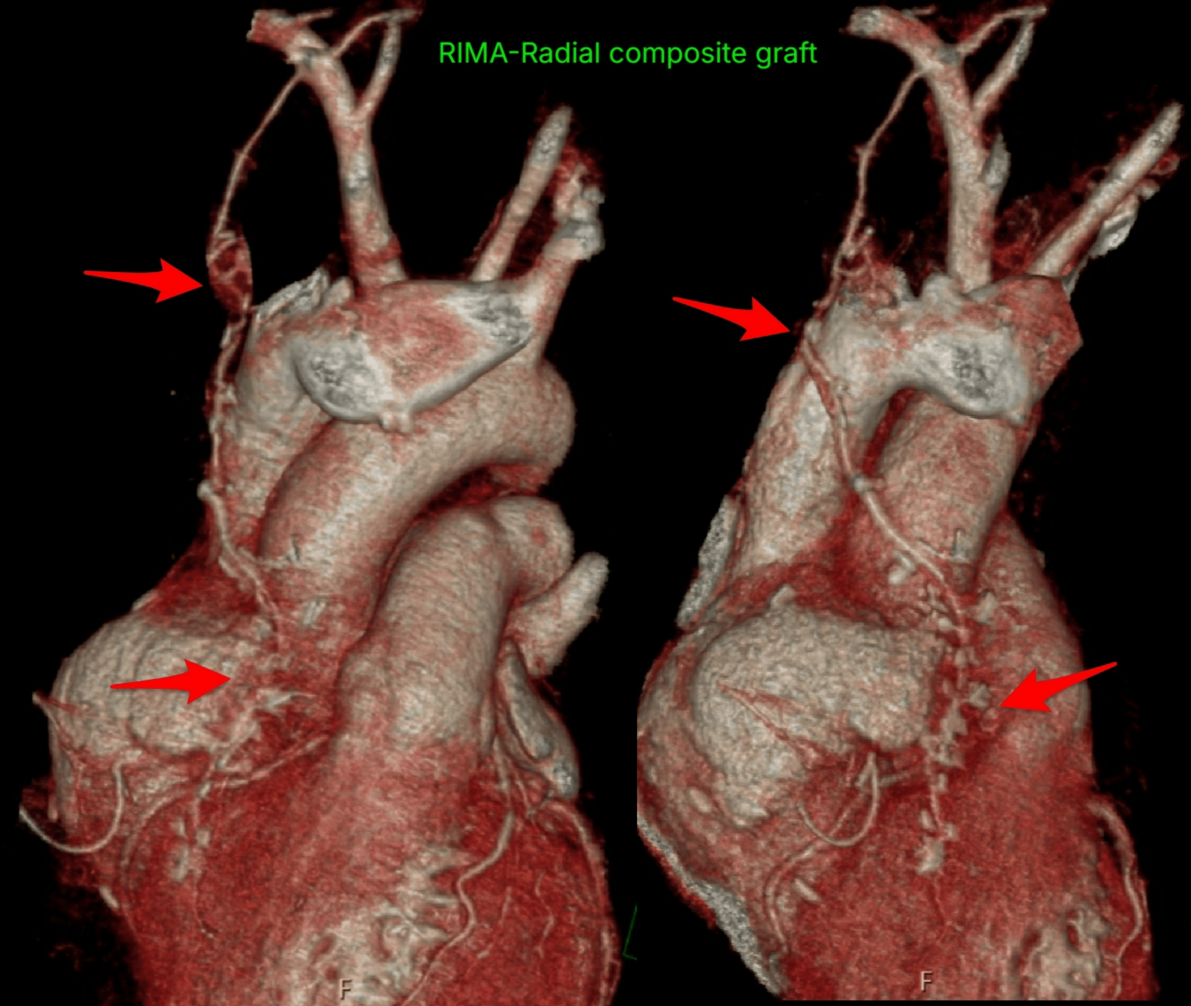
RIMA-radial composite graft anastomosed to posterior descending artery. RIMA: right internal mammary artery

RIMA was harvested and a RIMA-radial artery composite graft was constructed for achieving adequate conduit length. Peripheral cardiopulmonary bypass was instituted via right femoral artery and vein cannulation (Figure 2). The RCA was identified arising between the great vessels and traversing the right ventricular free wall in a dual RCA/early bifurcation before assuming a normal distal course. Using an octopus stabilizer, an end-to-side anastomosis of the composite graft to the mid-RCA was performed (Figure 3).

**Figure 4:**
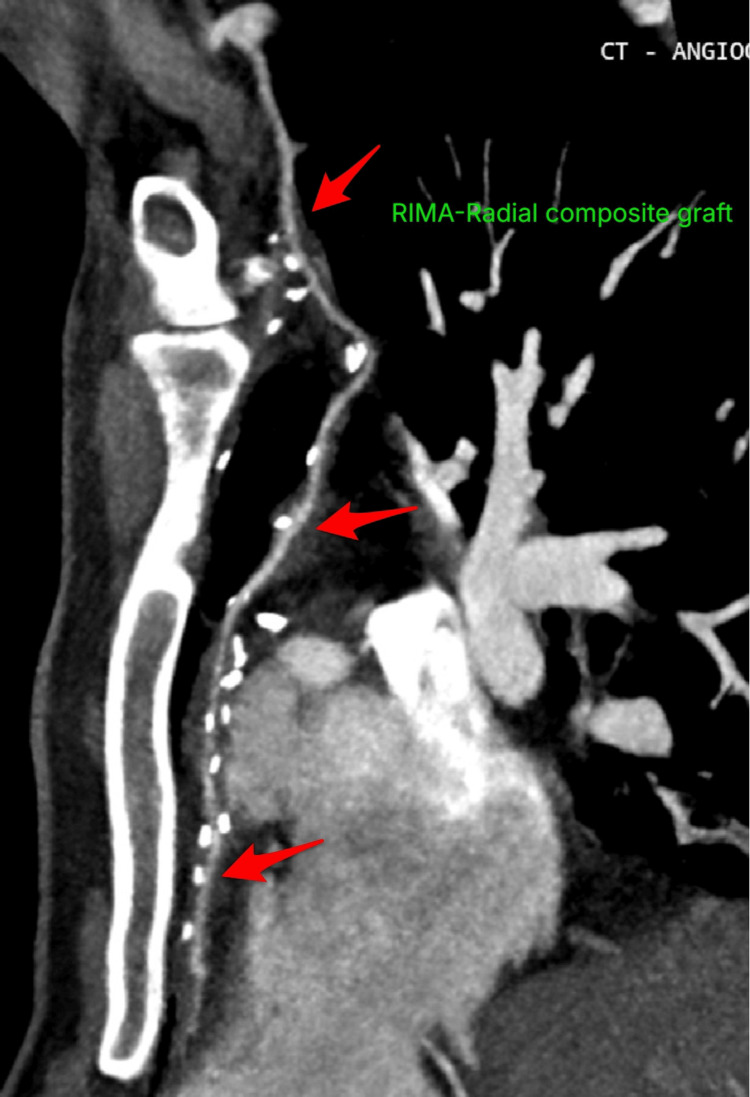
Coronal cut showing graft patency of the composite graft. RIMA: right internal mammary artery

**Figure 5:**
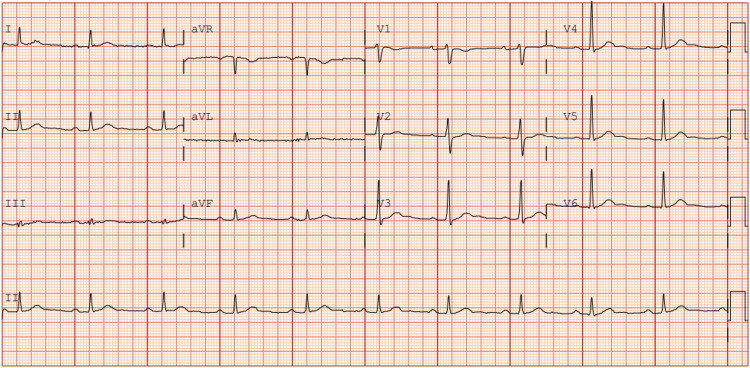
Follow-up electrocardiogram

The patient was weaned from cardiopulmonary bypass with low-dose noradrenaline support (0.05µg/kg/min). The proximal native RCA was ligated. No ischemic ECG changes were noted in the inferior leads, and no new regional wall motion abnormalities were observed on transesophageal echocardiogram. Transit time flowmetry was done to rule out native vessel competitive flow. Postoperative CTCA demonstrated a patent RIMA-radial composite graft to PDA with good distal opacification (Figure 4). The native LAD and LCx remained normal. There was no evidence of myocardial infarction. The patient had an uneventful recovery. He was extubated on postoperative day one and discharged from the intensive care unit on postoperative day two. At six-month follow-up, the patient remained completely asymptomatic with normal ECG findings (Figure 5) and preserved biventricular function with no structural abnormalities on transthoracic echocardiography. 

## Discussion

AAORCA with an interarterial course represents a high-risk congenital variant that can result in myocardial ischemia and SCD. The clinical significance of this anatomy has been well-established, with AAORCA representing a leading cause of exercise-related sudden death in young individuals [2]. AAORCA can undergo three different courses: (1) a high interarterial course between the aorta and pulmonary artery, (2) a hypoplastic anomalous orifice with shorter interarterial course, or (3) a low interarterial course between the aorta and right ventricular outflow tract.

The AAOCA management strategy, including surgical referral, depends mainly on patient symptoms and presence of high-risk features in coronary imaging. The American Association for Thoracic Surgery expert consensus guidelines published in 2017 support that individuals with AAOCA with symptoms (ischemic chest pain, syncope secondary to ventricular arrhythmia, or history of aborted SCD) should be activity-restricted and offered surgery (Class I, Level of Evidence B) [1].

While unroofing procedures have gained popularity for many cases, the presence of a short intramural segment and high-bifurcation pattern in our patient made this approach less favorable. Previous surgical series have demonstrated that unroofing may be associated with suboptimal outcomes when the intramural course is brief or when complex branching patterns exist [3,4]. In our patient as depicted above the origin of the RCA from the left sinus was distinct in a way that it had a normal opening with a short intramural course and an early high-bifurcation into a twin RCA system causing dual branching of the RCA with a further anteriorly placed anatomy.

The choice of surgical option as a right MIDCAB was primarily based on the high-bifurcation of the RCA branch. It was a patient preference to opt for minimal invasive surgery for early recovery after surgery. Hence it was decided to go ahead with a right MIDCAB with RIMA grafting. Since the length of the RIMA was not sufficient enough, the RIMA was extended with a radial extension to anastomose to the early PDA. Proximal vessel ligation was performed to eliminate competitive flow, a technique that has proven effective in similar cases [5].

The patient needed peripheral bypass support to prevent perioperative right ventricular dysfunction and the risk of arrythmias while tying off the proximal bigger distal bifurcation branch. We did not opt for the unroofing or root translocation in view of the short intramural course and the need for double-barrel ostium while translocating which can lead to grievous postoperative anastomotic complications and perioperative fatal myocardial infarctions. The patient was successfully weaned off peripheral bypass without perioperative ischemic events or right ventricular dysfunction.

## Conclusions

Our experience demonstrates that the right MIDCAB remains a viable option for complex AAORCA cases where anatomical repair may not be optimal. The excellent short-term outcomes, including complete symptom resolution and patent graft on follow-up imaging, support this minimally invasive approach for patients with challenging coronary anatomy. This case highlights the importance of individualized surgical planning based on specific anatomical variants, particularly when standard techniques such as unroofing are not feasible due to short intramural course and high-bifurcation patterns. Long-term surveillance will be necessary to assess graft durability and establish the role of this technique in the comprehensive management of complex AAORCA cases.
